# Unravelling the impact of the chromobox proteins in human cancers

**DOI:** 10.1038/s41419-025-07585-1

**Published:** 2025-04-02

**Authors:** Shabana Noreen, Nicla Simonelli, Rosaria Benedetti, Vincenzo Carafa, Michele Grieco, Concetta Ambrosino, Carmela Dell’Aversana, Angela Nebbioso, Mariarosaria Conte, Nunzio Del Gaudio, Lucia Altucci

**Affiliations:** 1 Department of Precision Medicine, University of Campania “Luigi Vanvitelli”, Vico L. De Crecchio 7, 80138 Naples, Italy; 2 UP Medical Epigenetics, AOU Vanvitelli, Naples, Italy; 3 Biogem Institute of Molecular and Genetic Biology, Ariano Irpino, Italy; 4 Department of Environmental, Biological and Pharmaceutical Sciences and Technologies, University of Campania “Luigi Vanvitelli”, Caserta, Italy; 5 Department of Medicine and Surgery, LUM University, Casamassima, BA Italy; 6 Institute of Experimental Endocrinology and Oncology “Gaetano Salvatore” (IEOS)-National Research Council (CNR), 80131 Naples, Italy; 7 Department of Life Sciences, Health, and Health Professions, Link Campus University, Via del Casale Di San Pio V 44, 00165 Rome, Italy

**Keywords:** Oncogenes, Epigenetics

## Abstract

Chromobox (CBX) proteins play a crucial role in regulating epigenetic processes. They are extensively involved in various biological processes, including embryonic development, stem cell maintenance, cell proliferation and apoptosis control. The disruption and malfunction of CBXs in cancer typically results in the interference or abnormal activation of developmental pathways, which facilitate the onset, growth, and advancement of cancer. This review initially introduces the physiological properties and functions of the CBXs. Subsequently, it examines the involvement of CBXs in different cancer types. Cancer hallmarks driven by CBXs are mediated through multiple mechanisms, including changes in gene expression patterns, epigenetic dysregulation of chromatin control, disruption of intracellular signaling and alterations in cell metabolism. The study also highlights novel potential anticancer therapeutics targeting CBXs in cancer. In this review we provide novel perspectives and a solid foundation for future investigations on CBXs as promising therapeutic targets for cancer treatment.

## Facts

- CBX proteins are crucial epigenetic interpreters that control a wide range of biological processes, including embryonic development, stem cell maintenance, cell proliferation, and apoptosis.
- Dysregulation of CBX proteins is implicated in cancer, where they contribute to tumor progression by epigenetically altering key pathways that promote oncogenesis.
- Novel therapeutic agents targeting CBX proteins are under development, showing promising preclinical results.

## Open questions

- What are the mechanisms driving the deregulation of CBXs in cancer?
- How do CBX aberrations affect the activity of PRCs in cancer, and to what extent?
- What are the interactions among the members of the CBX family under normal physiological and pathological conditions, and how do these interactions influence cancer progression and potential future therapeutic options?

## Introduction

Epigenetics regulates gene expression without changes in the underlying DNA sequence. It can activate or suppress gene expression in response to external influences, cell functions and developmental signals. Epigenetic regulation revolves around three essential components: *writers, readers, and erasers* [1].

Epigenetic *writers*, the enzymatic catalysts, play a pivotal role in adding chemical modifications, such as methyl or acetyl groups, to DNA or histone proteins. These chemical modifications can significantly influence the accessibility and functionality of genes. On the other hand, *readers* are responsible for identifying and interpreting these chemical marks, thereby converting them into specific cellular functions. Lastly, *erasers* are crucial for eliminating these marks, thereby enabling the reversal of epigenetic signs and potentially resetting gene expression patterns.

The interaction among *writers*, *readers*, *and erasers* establishes an intricate and subtle mechanism of gene control that can be passed down through cell divisions and, in certain instances, even across generations [2].

The epigenetic apparatus is essential for various biological activities, such as cell differentiation, embryonic development, and the body’s reaction to external stimuli [3]. For these reasons, epigenetics holds significant potential in medicine, with applications rapidly expanding. In cancer therapy, drugs targeting epigenetic regulators have shown reactivation of tumor-suppressing and/or silencing of cancer-promoting genes ultimately arresting cancer cell proliferation. Importantly, these therapies can reverse harmful gene expression changes, offering safer, more precise treatments and advancing innovative medical approaches [4].

The CBX family comprises eight members that are all characterized by a widely conserved domain structure called chromodomain whose function is to bind methylated histone residues. Despite this similarity, each CBX has a unique structure and plays specific functions that are cell-context specific. Among the CBXs, CBX2, CBX4, CBX6, CBX7 and CBX8 belong to the polycomb group proteins, while CBX1, CBX3 and CBX5 belong to heterochromatin protein 1 (HP1).

This review aims to offer a comprehensive overview of the various functions of the CBXs family and its involvement in advancing cancer and forming tumors. Specifically, we examine the mechanisms by which CBX proteins control gene expression, stem cell renewal, and other cellular processes that contribute to the genesis and advancement of cancer. We speculate on the clinical importance and predictive ability of CBXs in different forms of cancer. The study aims to thoroughly comprehend the many roles of CBXs in cancer biology and their potential targets for therapy and/or as prognostic indicators.

In the next paragraphs we will elucidate the physiological role of CBXs and their potential implication in human cancers. We will then specifically shed light on mechanisms by which CBXs control gene expression and cellular processes contributing to cancer development.

## Physiological role of the CBX proteins

### Chromobox proteins

CBXs form a family of eight epigenetic regulators, each characterized by a highly conserved chromobox domain (ChD) at the N-terminus. This domain enables CBXs to interact with histone modifications, such as H3K27me3 and H3K9me3, playing a crucial role in regulating gene expression across various biological processes, including stem cell pluripotency, cell cycle control, senescence, and cancer progression [5]. The CBX family is further divided into two subgroups based on differences in their C-terminal domains: the Polycomb group (PcG) family, which includes CBX2, CBX4, CBX6, CBX7, and CBX8, and the heterochromatin protein 1 (HP1) family, CBX1 (HP1β), CBX3 (HP1γ), and CBX5 (HP1α) [6]. Members of the PcG family possess a conserved polycomb repressor box (Pc box), which integrates them into cPRC1 complexes. In contrast, the HP1 family is defined by a single conserved chromoshadow domain (CSD), which facilitates their role in heterochromatin formation, independently from PRC1 complexes. In mammals, the ChD of CBX proteins belonging to of PcG family exhibit differential affinity for H3K27me3 and H3K9me3 [7]. For example, CBX4 demonstrates a preferential binding to H3K9me3, while CBX7 displays affinity for both H3K27me3 and H3K9me3. In contrast, CBX6 shows a considerably lower affinity for either modification [8]. CBX2 undergoes a phosphorylation-dependent mechanism, which further increases the binding affinity to H3K27me3 following the phosphorylation of serine 42 within its ChD by casein kinase 2 (CK2) [9]. Despite these variations, recognizing H3K27me3 remains a central regulatory mechanism due to its critical role in Polycomb repressive complex 1 (PRC1) localization and function [10]. PcG family CBXs are also distinguished by additional DNA-binding motifs, such as the AT-hook motif, found exclusively in CBX2, or AT-hook-like motif, present in other CBXs [11]. The AT-hook domain of CBX2 binds to AT-rich regions within the minor groove of DNA, mediating a CBX2 histone-independent DNA interaction [12]. On the contrary, the HP1 family primarily acts in heterochromatin formation [13]. The ChD of HP1 proteins shares homology with that of PcG CBXs and selectively binds to H3K9me2/3 [6]. This interaction allows HP1 proteins to recruit the H3K9 methyltransferase SUV39H1, which methylates adjacent H3K9 residues, thereby establishing a positive feedback loop by generating new binding sites for HP1 [14]. HP1 proteins also contain a conserved chromoshadow domain (CSD) in their C-terminal region. Although structurally similar to the ChD, the CSD promotes homo- and heterodimerization of HP1 proteins, a process distinct from the histone-binding function of the ChD [15].

### *PRC1 and PRC2*

CBX proteins, as integral components of polycomb group complexes, play a crucial role in epigenetic regulation, gene silencing, and chromatin remodeling.

Biochemical purification experiments in *Drosophila* enabled the classification of the Polycomb machinery into two principal complexes: Polycomb Repressive Complex 1 (PRC1) and Polycomb Repressive Complex 2 (PRC2) [16]. Both complexes possess histone-modifying activity that induces chromatin remodeling and compaction, thereby establishing a repressive chromatin state that effectively silences gene expression [17].

PRC1 primary mediates the monoubiquitination of histone H2A at lysine residues 117 and 118 (H2Ak117ub and H2Ak118ub) [18], while PRC2 catalyzes the mono-, di-, and trimethylation of histone H3 at lysine 27 (H3K27me1, H3K27me2, H3K27me3). The catalytic subunit RING1A/B interacts with accessory subunits to form at least eight distinct PRC1 complexes, categorized into ‘canonical’ PRC1 (cPRC1) and ‘non-canonical’ or ‘variant’ PRC1 (ncPRC1 or vPRC1), based on their discovery order and the specific subunit composition [19]. CBX proteins and either RYBP (RING1 and YY1 binding protein) or YAF2 (YY1-associated factor 2) mutually compete for binding to RING1A/B, thereby defining cPRC1 and ncPRC1 complexes respectively and influencing their composition, function, and molecular activity [19, 20]. PRC1 complexes are subdivided into six major groups (PRC1.1–PRC1.6) based on the specific Polycomb group Ring Finger protein (PCGF1–6). cPRC1 complexes typically incorporate PCGF2 (also known as MEL18) or PCGF4 (also known as BMI1), whereas ncPRC1 complexes can include any of the six PCGF proteins. Additionally, PRC1.2 and PRC1.4 can be further divided into canonical (cPRC1.2 and cPRC1.4) and non-canonical (ncPRC1.2 and ncPRC1.4) variants [19].

**Fig. 1:**
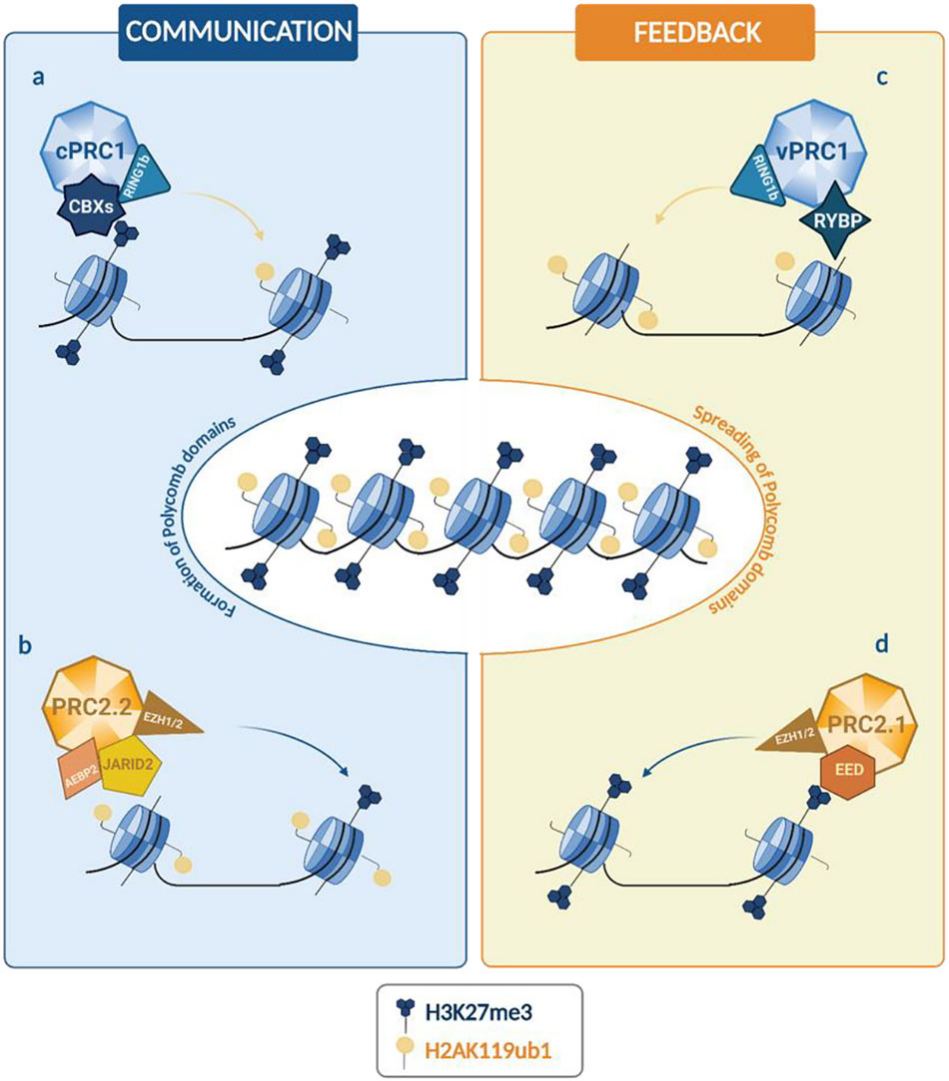
Communication and feedback mechanisms among PRCs. **a** H3K27me3, added by PRC2 variants, is recognized by the CBX subunit within cPRC1 complexes which contribute to H2AK119 ubiquitylation. **b** H2AK119 is subsequently recognized by the JARID2 and AEBP2 subunits of PRC2.2 **c** H2AK119ub1 can be also recognized by the RYBP subunit of vPRC1 complexes. **d** H3K27me3 can be recognized by the EED subunit of PRC2.1, which allosterically activates the methyltransferase activity of PRC2, enabling H3K27me3 propagation.

PRC2 catalyzes the mono-, di-, and tri-methylation of lysine 27 on histone H3 (H3K27me1, H3K27me2, H3K27me3), thereby maintaining transcriptional repression of target genes [21]. The PRC2 is arranged into two functional lobes interfaced by the SUZ12 (Suppressor of Zeste 12) subunit, which is involved in the stabilization of the complex, the modulation of its catalytic activity, and the determination of its localization [22, 23]. The catalytic lobe of PRC2 contains EZH2 or its paralog EZH1 (Enhancer of Zeste Homolog 2/1), along with the EED (Embryonic Ectoderm Development) subunit and the VEFS domain of SUZ12 [24]. The targeting and regulating domain primarily comprises the N-terminal portion of SUZ12 associated with RBBP4 or RBBP7 (Retinoblastoma Binding Protein 4/7) [25]. These proteins mutually interact with various subunits, leading to the classification of PRC2 into two distinct variants, PRC2.1 and PRC2.2, with distinct regulatory properties [25]. A summary of the defined subunit domains and known biochemical functions is provided in Tables 1 and 2. The combined footprint of H3K27me3 and H2AK119ub1 marks plays a crucial role in gene repression, acting synergically and enabling communication between PRC1 and PRC2 activities. These Polycomb chromatin domains cooperate to establish a fine-tuned regulatory mechanism (Fig. 1). Biochemical studies have revealed a functional link between PRC2.2 and H2AK119ub1 deposited by PRC1 [26]. Specifically, PRC2.2 engages with H2AK119ub1-marked nucleosomes through its JARID2 (via its ubiquitin-interacting motif) and AEBP2 (via its C2H2 zinc-finger domains) subunits [27]. These interactions stimulate the methyltransferase activity of PRC2.2, leading to the enrichment of H3K27me3 at these loci [28]. H3K27me3 acts as a recognition signal for CBX ChD, within cPRC1, binds H3K27me3, localizing the complex activity to sites previously marked by PRC2, thereby establishing a PRC2-dependent recruitment mechanism [29] (Fig. 1).

**Table 1:** Biochemical functions of PRC1 subunits.

PRC1	Subunit	Motif/domains	Function	Ref
Core	RING1A RING1B	N-terminal RING-type zinc Finger C-terminal RAWUL domain	E3 ubiquitin-ligase activity	[20]
cPRC1	CBX2 CBX4 CBX6 CBX7 CBX8	N-terminal Chromodomain AT hook (CBX2) or AT-like hook (CBX4,6,7,8) C-terminal Pc Box	Binding of H3K27me3 allowing cPRC1 binding to chromatin (H3K27me3 dependent)	[11]
cPRC1	PHC1 PHC2 PHC3	FCS-type zinc finger C-terminal Sterile alpha motif (SAM)	Facilitates heterotypic or homotypic interactions between PRC1 complexes	[160]
ncPRC1	RYBP YAF2	N-terminal zinc finger Yaf2/RYBP C-terminal binding motif	Binding to H2K119ub and promoting its propagation	[161]
ncPRC1	PCGF1 PCGF3 PCGF5 PCGF6	N-terminal RING-type zinc Finger C-terminal RAWUL domain (except PCGF6)	Formation of RING-domain mediated heterodimer with RING1 protein	[162]
cPRC1/ nCPRC1	PGF2 or MEL18 PGF4 or BMI1	N-terminal RING-type zinc finger C-terminal RAWUL domain	Stability of the core and enhancing of catalytic activity	[163]

**Table 2:** Biochemical function of PRC2 subunits.

PRC2	Subunit	Motif/domains	function	Ref
Core	EZH1 EZH2	N-terminal WD repeat binding domain SANT domain CXC domain C-terminal SET domain	Histone methyltransferase (H3K27me3)	[164]
Core	EED	WD-40 repeats	Allosteric activation of EZH1/EZH2	[165]
Core	SUZ12	C-terminal VEFS Box	Stability of core	[22]
Core	RBBP4 RBBP7	WD40 repeats	Binding to nucleosome	[166]
PRC2.1	PCL1 or PHF1 PCL2 or MTF2 PCL3 or PHF19	N-terminal TUDOR domain PHD-type zinc finger C-terminal Polycomb like MTF2 factor 2 domain	Binding to H3K36me3 recruiting PRC2.1	[167]
PRC2.1	EPOP	BC box	Connecting Elonging BC to PRC2 core	[168]
PRC2.1	PALI1	Helix-turn-helix DNA binding domain	Facilitating chromatin binding and enhancing PRC2 activity	[169]
PRC2.2	JARID2	ARID DNA Binding domain Jmj domain Zinc finger	Enhancement of PRC2 methyltransferase activity and recruitment of the complex to H2AK119ub1	[170]
PRC2.2	AEBP2	Zinc finger	Enhancement of PRC2 methyltransferase activity and recruitment of the complex to H2AK119ub1	[27]
CATACOMB-containing PRC2	CATACOMB	Serine rich domain	Allosteric inhibition of PRC2	[171]

This interdependence is further reinforced by a self-propagating mechanism where PRC variants expand their catalytic marks to adjacent nucleosomes. Specifically, RYBP subunit (via its N-terminal zinc-finger domain) of ncPRC1 recognize H2AK119ub1, facilitating, by the support of histone H1, the extension of this mark to adjacent nucleosomes [30] (Fig. 1). Concurrently, PRC2 binds H3K27me3-containing nucleosomes through its EED subunit (via the WD40-repeat domain), positioning the SET domain of EZH2 to methylate unmodified H3 tails on neighboring nucleosomes [31]. Optimal methyltransferase activity by PRC2 also requires a short DNA linker length between nucleosomes, which facilitates efficient chromatin condensation and gene silencing [32].

### Biological role of CBXs

The CBXs are essential in several biological processes, such as embryonic development, maintenance of stem cell characteristics, cell proliferation, DNA damage response, and X-chromosome inactivation [33].

Among these, CBX7 is key in preventing premature differentiation in stem cells, particularly in hematopoietic stem and progenitor cells (HSPCs) [34]. Conversely, CBX6 and CBX4 play a key role in balancing embryonic stem cell’s pluripotency and differentiation by suppressing the expression of the pluripotency genes SOX2 and NANOG, with their stability being facilitated by USP26 activity, which prevents their degradation during differentiation [35]. CBX2 perturbations in human hematopoietic progenitors impair proliferation and reduce colony-forming potential in vivo [36]. In contrast, CBX2 overexpression (OE) induces lymphoid and myeloid differentiation [37]. CBX8 knockdown (KD) inhibits colony formation in HSPCs, whereas CBX8-OE enhances colony formation in vitro and cell proliferation in vivo [34]. CBX1 helps preserve pluripotency [38], while CBX3 balances pluripotency, differentiation, and early developmental stages of pluripotent stem cells [38]. Knockout (KO) mouse studies have shown that silencing CBXs causes significant developmental defects, underscoring its critical role in embryogenesis and tissue formation. Specifically, CBX2 KO mice exhibit premature activation of HOX gene expression [39] and affect the sex determination and development of reproductive organs [40]. CBX4 is essential for proper thymus and skin development [41]. CBX7, conversely, is necessary for neurogenesis, as its KD promotes axon growth in both embryonic cortical neurons and adult dorsal root ganglion (DRG) neurons [42]. CBX1 is critically required for neurogenesis and brain development too [43]. In contrast, CBX3 and CBX5 are essential for kidney development, particularly in mediating kidney branching and renal morphogenesis [44].

Several evidence from cell-based studies indicate that CBXs are essential in regulating multiple cell cycle checkpoint mechanisms [45]. CBX2 regulates genes involved in cell cycle progression through the PI3K/AKT and Hippo/YAP signaling pathways [46]. CBX4 also regulates cell cycle progression during the G2/M phase, influencing mitotic entry, [47] and delays the G1/S transition by reducing proliferating cell nuclear antigen (PCNA), cyclin E2 (CCNE2) and CDK2, cyclin A2 (CCNA2) levels, while upregulating CDKN2A/p16 expression [48]. CBX6 can inhibit cell division by downregulating cyclins CCND1 and CCNE1, indirectly promoting the upregulation of CDK inhibitors CDKN1A (p21), CDKN1B (p27), and CDK2B/p15 [49]. Multiple studies have also highlighted CBX7’s role in negatively controlling the G1/S transition by repressing cyclin E1 (CCNE1) expression through interaction with histone deacetylase 2 (HDAC2) [50].CBX8 promotes cell growth by positively regulating YBX1 and Cyclin D1 (CCND1) [51]. CBX1 promotes cell proliferation by modulating the canonical Wnt/β-Catenin signaling pathway [52], while CBX3 enhances cell cycle progression through the G2/M phase, preventing apoptosis and G0/G1 arrest [53]. Although the role of CBX5 in cell growth is less well studied, it has been linked to defective DNA repair mechanisms [54]. Summarizing, all CBX proteins play essential and diverse roles in various biological processes. Specifically, CBX7 prevents premature differentiation in stem cells and promotes neurogenesis, while CBX6 and CBX4 regulate pluripotency and differentiation. CBX2 is involved in modulating hematopoietic progenitor proliferation and differentiation and CBX8 affects colony formation and cell growth. CBX1 maintains pluripotency and supports neurogenesis, whereas CBX3 is crucial for balancing pluripotency, differentiation, and early developmental processes. Additionally, CBX5 plays a role in DNA repair.

**Fig. 2:**
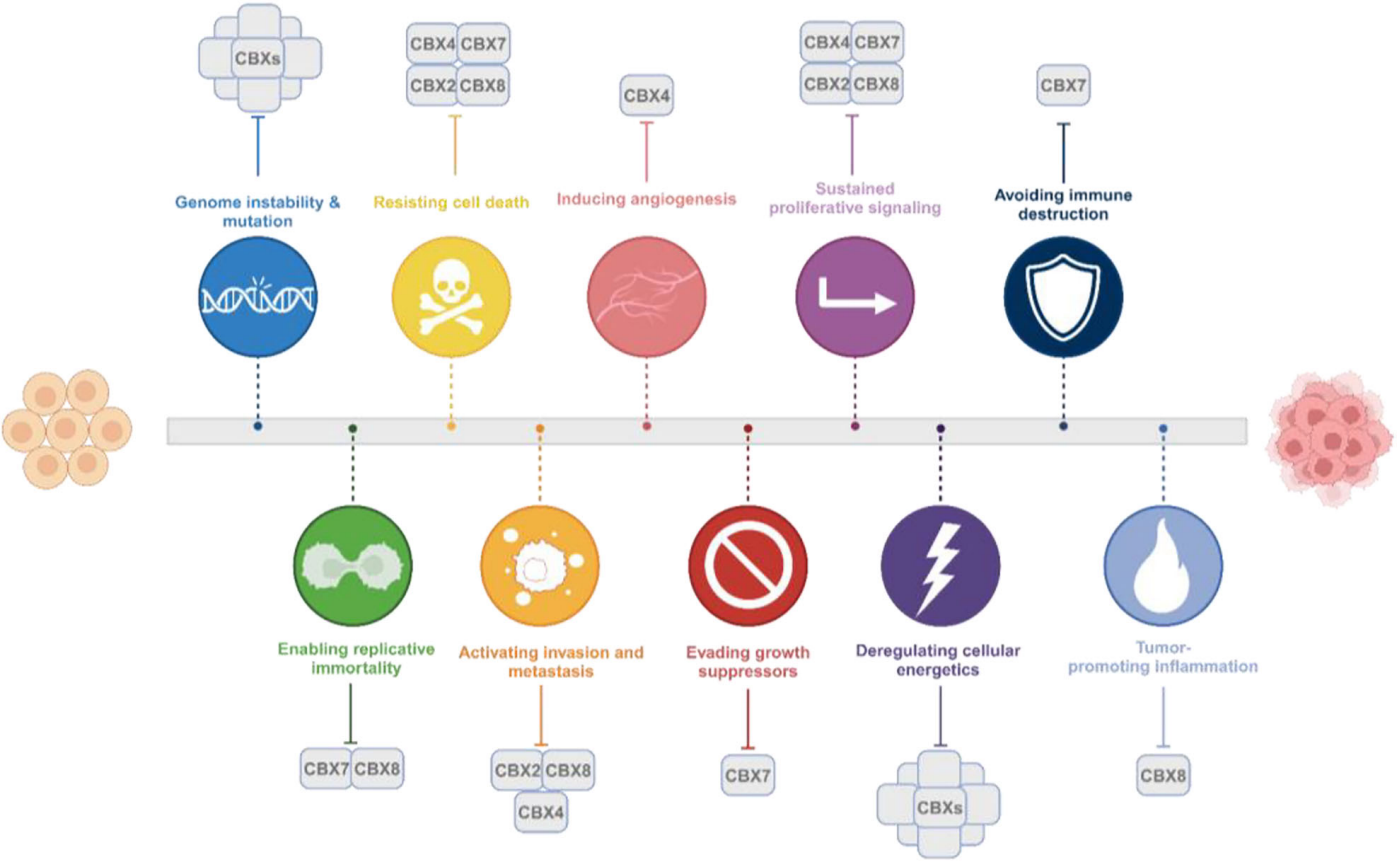
Roles of CBXs in the Hallmarks of Cancer. CBX proteins play crucial roles in several hallmarks of cancer through their functions as epigenetic regulators. CBX2, CBX4, CBX7, and CBX8 promote cell proliferation in various cancers by preventing cell death. CBX2, CBX4, and CBX8 are also involved in activating invasion and metastasis processes, while CBX4 may play a role in inducing angiogenesis. CBX7 can modulate tumor suppressor pathways and enhance anti-tumor immunity, whereas CBX8 contributes to tumor progression.

The following sections elucidate in a schematic view the molecular implications of the CBXs in tumorigenesis, organized by cancer type, focusing on their influence on gene expression, cellular processes, and contributions to cancer hallmarks (Fig. 2).

## Molecular role of CBX2 in cancer

Among the CBX family members, CBX2 stands out for its significant impact on cancer biology [6]. Dysregulation of CBX2 can disrupt essential epigenetic controls, contributing to tumor initiation and progression by affecting cell proliferation and survival pathways [55].

### Triple-negative breast cancer

CBX2 plays a crucial role in triple-negative breast cancer (TNBC) progression through its regulation of vital oncogenic pathways [56]. In vitro studies have shown that CBX2 can promote tumor growth and invasiveness by activating the PI3K/AKT pathway, a signaling cascade inducing phosphatidylinositol 3-kinase (PI3K), which phosphorylates phosphatidylinositol 4,5-bisphosphate (PIP2) to generate PIP3. PIP3 then activates AKT, a serine/threonine kinase that phosphorylates numerous downstream targets involved in cell survival, proliferation, and metabolism. In TNBC, the PI3K/AKT pathway is frequently hyperactivated due to various mechanisms, including mutations in PIK3CA (encoding the catalytic subunit of PI3K) or loss of function of PTEN (a negative regulator of the pathway). This hyperactivation contributes to the aggressive nature of TNBC [57]. CBX2 has been also identified as a critical regulator that inhibits the DREAM complex (comprising dimerization partner, RB-Like proteins, E2F family members, and multi-vulval class B proteins), leading to dysregulated cell cycle progression and unchecked cellular proliferation of BC. Additionally, CBX2 enhances mTORC1 (mammalian target of rapamycin complex 1) signaling, which drives cellular growth and shifts metabolic pathways in BC cell lines [58]. Of note, CBX2 was found to be overexpressed in human breast tumors compared to normal tissues, and the expression of CBX2 was inversely correlated with the patient’s prognosis, suggesting that high levels of CBX2 are associated with a worse prognosis and could serve as an important prognostic biomarker for patients with breast cancer (BC). Interestingly, elevated CBX2 expression is significantly associated with key clinical features, such as positive lymph node metastasis, increased tumor size, and HER-2 positivity [59].

### Gastric cancer

In gastric cancer (GC) cell lines, high CBX2 expression promotes proliferation, invasion, and migration by activating the YAP/β-catenin pathway [60]. Mechanistically, CBX2 inhibition prevents Yes-associated protein (YAP) translocation to the nucleus and subsequent phosphorylation, suppressing β-catenin signaling. This highlights a critical interplay between the YAP and β-catenin pathways, where CBX2 promotes GC progression by facilitating YAP’s activation of β-catenin [60].

### Hepatocellular carcinoma

In hepatocellular carcinoma (HCC) cells, CBX2 downregulation slows proliferation and induces apoptosis [61]. This mainly occurs through a mechanism involving the suppression of Wilms’ tumor protein 1-interacting protein (WTIP) expression. This leads to the activation of the Hippo pathway, phosphorylation and inactivation of YAP, and ultimately reduced transcriptional activity of YAP [62].

### Lung adenocarcinoma

CBX2 has emerged as a critical oncogenic driver in lung adenocarcinoma (LUAD). Together with EZH2, it promotes tumor growth and metastasis through epigenetic mechanisms. CBX2 KD significantly inhibited LUAD cell growth and metastasis in vitro and in vivo [63]. Notably, CBX2 is significantly upregulated in LUAD tissues, and elevated levels of CBX2 expression are correlated with poor patient prognosis [64]. Interestingly, CBX2 KD significantly hampers LUAD cell growth and metastasis in cancer cells in vivo. However, the simultaneous inhibition of CBX2 and EZH2 exhibits a more potent suppressive effect on LUAD cells than individually targeting either protein [65].

### Colorectal cancer

CBX2 has been found overexpressed in colorectal cancer (CRC) and associated with poor prognosis [66, 67]. Specifically, it promotes proliferation, migration, and invasion of CRC cell lines while inhibiting apoptosis through two independent mechanisms [67]. First, CBX2 directly binds to the promoter regions of cyclin-dependent kinase inhibitor (CDKI) genes p21 and p27, facilitating H2AK119ub1 deposition and transcriptional repression. CBX2 KD reduces H2AK119ub1 levels near these promoters, increasing p21 and p27 expression and inducing cell cycle arrest [68]. Secondly, CBX2 positively regulates genes of the MAPK signaling pathway, enhancing cancer cell proliferation and promoting cell migration [69].

### Prostate cancer

CBX2 is frequently upregulated in metastatic prostate cancer (mPCa), and its expression correlates with poor clinical outcomes. Silencing CBX2 results in reduced cell viability and increased apoptosis in mPCa cell lines, suggesting its critical role in regulating genes involved in cell proliferation and metastasis. Mechanistically, CBX2 suppression in mPCa cell lines induces apoptosis by downregulating key cell cycle regulators, including Aurora kinases A and B (AURKA and AURKB), cyclin B1, Ki-67 (MKI67), CDK1, and CDC25A, all of which are interconnected and essential for mPCa cell survival. These findings position CBX2 as a promising therapeutic target for advanced prostate cancer [70, 71].

### Ovarian cancer

CBX2 promotes cancer cell proliferation and tumor progression and is associated with the survival of high-grade serous ovarian cancer (HGSOC) patients. Loss of CBX2 destabilizes genomic integrity, inducing cell death. Interestingly, Wnt/β-catenin activation has been identified as a novel mechanism that allows cells to bypass death despite complete CBX2 loss [72]. Functional analyses reveal that CBX2 regulates genes involved in ovarian function, such as folliculogenesis and steroidogenesis. Additionally, CBX2 is a key regulator of genes associated with conditions like polycystic ovary syndrome, premature ovarian failure, and pituitary deficiencies [73, 74].

In addition, cell-based experiments highlighted that Circ_0061140, a circular RNA, acts as a molecular sponge for miR-136 in ovarian cancer (OC) cells, preventing miR-136 from binding to and regulating CBX2. This interaction increases CBX2 expression, ultimately promoting cancer cell proliferation, migration, and invasion [73]. These findings highlight the role of the Wnt/β-catenin pathway, CBX2, microRNAs, and circular RNAs in the progression of OC cells, suggesting the formation of a molecular axis for therapeutic intervention [75].

### Hematological disorders

In Hematological malignancies, CBX2 is often overexpressed and plays a crucial role in driving cancer cell progression. This is achieved through its interaction with key oncogenic pathways that inhibit differentiation and accelerate cell cycle progression [76]. Mechanistically, in acute myeloid leukemia (AML) cell lines, CBX2 KD alters the expression of genes within the p38 MAPK pathway by inducing chromatin reorganization at these regulatory regions, thereby affecting AML cell survival. Interestingly, CBX2 activity also maintains the epigenetic landscape required for LSC function [77].

## Molecular role of CBX4 in cancer

CBX4 plays critical roles in epigenetic regulation, including transcriptional repression and DNA damage response through its chromatin binding and SUMO E3 ligase activity. It is involved in various biological processes such as growth, senescence, and cancer progression, with its expression levels impacting tumorigenesis and prognosis in several cancers [78].

### Breast cancer

CBX4 is overexpressed in BC and functions as an oncogene by downregulating miR-137, which in turn enhances the activation of the Notch1 signaling pathway promoting epithelial-mesenchymal transition (EMT), invasion, and growth of BC cell lines [79]. Notch1-induced EMT reduces E-cadherin expression, a hallmark of EMT, which contributes to increased cancer cell motility and invasiveness [80]. This interconnected pathway represents a potential therapeutic target, where interventions aimed at any step could potentially disrupt the pro-metastatic effects of BC [81].

### Gastric cancer

CBX4 plays a critical role in GC by modulating histone modifications [82]. It recruits histone demethylases to erase the repressive H3K27me3 mark, facilitating its replacement with the active H3K4me3 mark, which is associated with gene activation [60]. This epigenetic modification leads to the upregulation of cancer-associated genes such as CDC20, thereby enhancing GC cell lines proliferation, migration, and metastasis [83]. Targeting CBX4 could reverse these oncogenic effects, offering a therapeutic strategy for GC [84].

### Hepatocellular carcinoma

In HCC cell lines, CBX4 has been found to mediate the ubiquitination and degradation of hypoxia-inducible factor-1 alpha (HIF-1α), reducing angiogenesis and glucose metabolism-related gene expression [85].

Further, CBX4-mediated regulation of miR-424 downregulates E2F7 and CDK1, blocking cell cycle progression and proliferation in HCC cells [86]. miR-424 also inhibits YAP1 through Hippo pathway modulation, reducing oncogenic signaling and stem cell-like traits in HCC cell lines and in vivo xenograft mouse models [86]. In a recent study, CBX4 was also identified as an independent prognostic marker for HCC. In patients with low CBX4 expression, treatments like transarterial embolization (TAE) and postoperative transarterial chemoembolization (TACE) do not significantly affect overall survival. However, high CBX4 levels are associated with improved survival following TACE, while TAE may reduce survival. This indicates that CBX4 can guide postoperative TACE decisions. Additionally, experimental data show that CBX4 overexpression increases doxorubicin-induced cytotoxicity in HCC cells [87].

### Lung adenocarcinoma

Overexpression of CBX4 in LUAD activates the Wnt/β-catenin pathway by stabilizing β-catenin preventing its phosphorylation and degradation [88]. This stabilization allows β-catenin to accumulate in the cytoplasm and translocate to the nucleus, where it triggers gene transcription essential for cell development and viability, leading to increased proliferation and invasion of LUAD cell lines [88]. Conversely, CBX4 KD reduces the synthesis of cyclin-dependent kinase 2 (CDK2) and cyclin E, halting cell cycle progression at the G0/G1 phase. CBX4 also regulates the expression of BMI-1, a key factor in cellular proliferation and migration in vivo. Consequently, CBX4 KD leads to reduced BMI-1 levels, which correlates with decreased proliferation and migration of cancer cell lines [89]. Interestingly, a recent study shows that CBX4 promotes LUAD growth by enhancing phosphoglycerate dehydrogenase (PHGDH) expression and serine biosynthesis, while simultaneously inhibiting metastasis by suppressing Zinc Finger E-box-binding Homeobox 2 (ZEB2). CBX4 enhances PHGDH expression through interaction with GCN5, increasing histone acetylation, and represses ZEB2 by recruiting cPRC1 to mark its promoter. These findings highlight CBX4’s dual role in LUAD progression revealing its dual regulatory functions through specific epigenetic interactions and suggest new therapeutic avenues [90].

### Colorectal cancer

In CRC, CBX4 acts as a tumor suppressor by interacting with histone deacetylase 3 (HDAC3) to repress the expression of the (runt-related transcription factor 2) RUNX2 gene, which activity promotes metastasis in CRC cell lines and mice model [91]. This repression is mediated by CBX4, which recruits HDAC3 to the RUNX2 promoter, maintaining a deacetylated state of histone H3K27 and suppressing RUNX2 expression. High CBX4 expression and low RUNX2 levels are associated with improved survival in colorectal cancer patients, suggesting that enhancing the CBX4-HDAC3 axis could serve as a potential therapeutic strategy for managing metastasis in colorectal cancer [92].

## Molecular role of CBX6 in cancer

CBX6 participates in mechanisms of chromatin regulation and is critical in cancer biology. It mediates transcriptional repression by binding to methylated histone tails, affecting gene expression and chromatin structure [93].

### Breast cancer

The transcriptional suppression of the CBX6 gene in MCF-7 BC cell lines is mediated by EZH2, which enhances H3K27me3 at the CBX6 promoter region [49]. This suggests a negative feedback loop where increased EZH2 protein levels may inhibit CBX6 expression, allowing BC cells to bypass G0/G1 cell cycle arrest, a mechanism usually activated in response to stress, DNA damage, or nutrient scarcity [94]. Consequently, this suppression facilitates continuous cellular proliferation and tumor progression [95].

### Hepatocellular carcinoma

CBX6 has been identified as a potential prognostic biomarker and therapeutic target of HCC [96]. In this tumor, CBX6 is overexpressed and correlates with poor prognosis and increased tumor proliferation [97]. Mechanistically, CBX6 promotes tumor growth through the S100A9/NF-κB/MAPK signaling pathway and contributes to metastasis of HCC cell lines and in vivo via transcription factors such as Snail and Zeb1 that mediate EMT [96]. Recently Zheng et al. demonstrated that elevated expression levels of CBX6 correlate with poorer prognostic outcomes in patients diagnosed with HCC [98].

### Hematological disorders

CBX6 may act as a tumor suppressor in chronic myeloid leukemia (CML), as its expression levels are low in untreated CML patients but increase during treatment with tyrosine kinase inhibitors (TKIs) used to manage the disease. This suggests that CBX6 might contribute to suppressing leukemic cell proliferation or survival, potentially offering a therapeutic target or biomarker for treatment response in CML [6].

## Molecular role of CBX7 in cancer

CBX7 is a multifaceted protein that plays critical roles in physiological processes and pathological conditions, particularly cancer [99]. Its complex functions make it a critical target for further research on cancer biology and regenerative medicine [100].

### Breast cancer

n BC patients, elevated expression levels of CBX7 were associated with improved survival outcomes. However, this upregulation also correlated with a poorer prognosis specifically in patients receiving either tamoxifen monotherapy or adjuvant chemotherapy make its correlation with prognosis and survival still controversial [101].

Experiments performed in BC cells showed that the transcriptional activity of CBX7 gene is suppressed by the high mobility group AT-hook 1 (HMGA1) protein. It is a widespread phenomenon observed in various cancer types, including BC MCF7 cell lines [102]. This interaction is significant because CBX7 typically functions as a tumor suppressor, while HMGA1 promotes tumor progression. By downregulating CBX7, HMGA1 contributes to cancer development by altering gene expression in cell cycle regulation, migration, and invasion of [103]. In addition, CBX7 negatively regulates miR-181b expression, which generally inhibits genes crucial for BC advancement. Conversely, CBX7 upregulates Dickkopf-1 (DKK-1) in CD44( + )/CD24(-)/ESA(+) breast stem-like cells and mouse models, which inhibit the Wnt/β-catenin pathway by trapping Frizzled receptors [104]. This inhibition attenuates dysregulated Wnt/β-catenin signaling, decreasing BC’s cell proliferation and tumorigenic potential [105].

### Gastric cancer

In GC, CBX7 is overexpressed and acts as an oncogene by enhancing cancer stem cell-like properties via the AKT-NF-κB-miR-21 pathway and downregulating the tumor suppressor p16, which promotes proliferation, invasion, and chemotherapy resistance in gastric cancer cell lines, consistent results were similarly obtained in mouse models [106].

### Pancreatic cancer

In pancreatic ductal adenocarcinoma **(**PDAC), CBX7 inhibits tumor progression by upregulating PTEN (Phosphatase and Tensin Homolog) expression, thereby negatively regulating the PI3K/AKT signaling pathway in Panc-1 and MIA PaCa-2 cell lines, as well as in nude mice [107].

### Prostate cancer

The Circular RNA Golgi phosphoprotein 3 (circ-GOLPH3) forms a complex with CBX7, influencing gene expression related to cell proliferation and cycle progression in prostate cancer (PCa) cell lines [108]. This interaction enhances CBX7’s stability and activity, upregulating genes that promote cell proliferation [109]. In cell line models of PCa, for example, CBX7 disrupts normal cell cycle regulation by repressing the tumor suppressor genes p16INK4A and p14ARF, thereby accelerating cancer progression and contributing to uncontrolled cell growth [110].

Additionally, CBX7 can inhibit TWIST1 by binding to the E-box sequence, which reduces the metastatic potential and tumorigenicity of secondary epithelial ovarian cancer in vitro and in vivo [111]. Further, CBX7 has been demonstrated to render TWIST-1 transcriptionally inactive during the mesenchymal-epithelial transition (MET) in ovarian cancer. Subsequently, a subclassification of OC was proposed, incorporating the expression levels of both CBX7 and TWIST-1. This subclassification serves as a predictive tool for clinical outcomes and patient prognosis in OC [111].

### Hematological disorders

CBX7 plays a dual role in hematological malignancies, acting as an oncogene in follicular lymphoma and a potential tumor suppressor in CML [112]. In follicular lymphoma, CBX7 is upregulated and contributes to lymphomagenesis and leukemogenesis, with its overexpression in hematopoietic stem cells driving the development of these cancers. Conversely, in CML, CBX7 expression is low in untreated cases but increases during TKI therapy, correlating with better treatment outcomes [113]. Patients exhibiting high CBX7 levels tend to achieve a faster complete hematologic response than those with lower expression, suggesting its role as a favorable prognostic marker in CML [114]. This dual role might be explained due to different cancer-associated backgrounds. In addition, CBX7 has been found to regulate the self-renewal of human hematopoietic stem and progenitor cells (HSPCs) and enhances their multi-lineage engraftment and myelopoiesis upon overexpression. It disrupts pathways involved in differentiation, DNA maintenance, and the cell cycle, and is upregulated in AML. Targeting CBX7 inhibits cell proliferation and promotes differentiation. Mass spectrometry identified interactions between CBX7 and non-histone proteins, including H3K9 methyltransferases SETDB1, EHMT1, and EHMT2. Depleting SETDB1 in AML cells mimics CBX7 repression, highlighting its regulatory role both in vitro and in vivo [34].

## Molecular role of CBX8 in Cancer

CBX8 binds to methylated histones, particularly H3K27me3, to repress developmental genes and maintain cellular identity during differentiation and development [115].

### Breast cancer

CBX8 plays a critical role in the progression of BC by modulating the epigenetic regulation of key signaling pathways. It is overexpressed in primary BC, and its high expression levels are correlated with poor patient outcomes [116]. CBX8 promotes tumor growth by maintaining a stem cell-like gene expression profile in BC cells, primarily through activation of the Notch signaling pathway, which is crucial for mammary development and tumorigenesis. Additionally, CBX8 sustains the transcriptionally active histone modification H3K4me3 at the promoters of Notch pathway-related genes and interacts with WDR5, a component of the H3K4 methyltransferase complex, thereby amplifying its role in the tumor progression of breast cancer cell lines. CBX8 also acts as a key regulator of mammary carcinoma in vivo [117].

### Hepatocellular carcinoma

CBX8 is overexpressed in HCC tissues compared to normal tissues, correlating with aggressive tumor behavior and shorter survival times. Mechanistically, CBX8 interacts with Y-box binding protein 1 (YBX1) to regulate the cell cycle through modulation of Cyclin D1 expression, thereby promoting proliferation in SK-Hep-1, Huh7, and MHCC-97H cells and supporting tumor development in mouse models [118]. Additionally, CBX8 activates the AKT/β-catenin signaling pathway by upregulating EGR1 and miR-365-3p, further driving tumor progression and metastasis in HCC cell lines and in vivo [119].

### Lung adenocarcinoma

CBX8 promotes tumor growth and metastasis capacity of lung adenocarcinoma cell lines and mouse models through the transcriptional repression of genes such as CDKN2C and SCEL, which are involved in cell cycle regulation and cellular adhesion [120].

CBX8 upregulation in LUAD has been associated with increased cancer cell invasion and migration, likely through pathways involving WNK2 and MMP2, which promote metastasis in 8- to 10-week-old NSG mice [121].

### Ovarian cancer

In ovarian cancer (OC), CBX8 promotes tumor growth and metastasis by interacting with acetyltransferases inhibitor subunit SET [122]. This interaction represses tumor suppressor gene SUSD2 expression by establishing H2AK119ub1 marks and preventing histone H3 acetylation, facilitating cancer cell proliferation and migration. Evaluating the effect of CBX8 KD on ovarian carcinoma growth and metastasis using subcutaneous and abdominal metastatic xenograft mouse models [122].

## Molecular role of HP1 family in Cancer

The HP1 gene family is crucial for chromatin architecture and regulation [123]. Specifically, CBX1 and CBX3 play a crucial role in heterochromatin formation, gene silencing, and DNA damage response and have been implicated in various cellular processes, including cell cycle regulation and cancer progression [124]. CBX5 is highly concentrated in heterochromatin and is significantly involved in physiological and pathological responses, such as controlling cell differentiation, proliferation, and death [125]. It connects with methylated lysine on position 9 of H3 (H3K9me), forming a transcriptional repressor complex that hinders gene transcription [126].

### Breast cancer

CBX1 functions as an oncogene in BC [94]. It is highly expressed in aggressive subtypes, such as TNBC, and is associated with poor prognosis, including shorter Relapse-Free Survival (RFS) and Overall Survival (OS) [95]. Additionally, CBX1 was found to be linked to chemoresistance in estrogen receptor-positive (ER + ) BC patients undergoing adjuvant chemotherapy. Recently, Young-Ho and colleagues discovered that breast cancer cells with depleted HP1β exhibit heightened sensitivity to PARP inhibitors. This reduction in HP1β levels may represent a valuable predictive biomarker for chemotherapy response [127].

CBX3 also significantly promotes BC cell proliferation, migration, and invasion by activating the ERK signaling axis and genes associated with EMT [128]. The ERK signaling pathway, which includes proteins such as mitogen-activated protein kinase (MEK) and ERK and is directly influenced by CBX3, highlighting the importance of CBX3 in breast cancer progression [94]. In BC, the crosstalk among CBX5 and E2F5 has revealed a nuanced regulatory mechanism [129]. Contrary to initial expectations, E2F5 intake leads to CBX5 upregulation in invasive BC cell lines, suggesting that E2F5 typically inhibits CBX5 expression. Reduced E2F5 levels increase CBX5 expression, which may inhibit tumor growth by promoting gene silencing through H3K27me3 binding [129]. Additionally, ubiquitinated CBX5 is attracted to chromatin regions rich in ncRNA, enhancing DNA damage and potentially triggering cell death or senescence. Interestingly, with their higher ncRNA levels and increased susceptibility to DNA damage, BC cells may be more sensitive to chemotherapy [130].

### Gastric cancer

CBX1 is significantly upregulated in GC tissues compared to normal gastric mucosa. Its overexpression correlates with advanced tumor stage, lymph node metastasis, and poor prognosis [131]. CBX1 promotes cancer proliferation by interacting with SUV39H1 to enhance H3K9 trimethylation and subsequent transcriptional silencing of tumor suppressor genes [132]. Interestingly, CBX1 has been shown to interact with the long non-coding RNA HOXA11-AS to promote gastric cancer cell lines proliferation and metastasis through the Wnt/β-catenin signaling pathway [133].

CBX3 is also highly expressed in gastric cancer tissues and cell lines. Its upregulation is associated with increased tumor size, advanced TNM stage, and lymph node metastasis [134]. At the molecular level, CBX3 promotes gastric cancer cell proliferation and invasion by regulating the expression of cell cycle-related genes such as CCND1 and CDK4. CBX3 interacts with the histone demethylase KDM4A to modulate H3K9 methylation levels and activate oncogenic pathways in GC cell lines [135, 136].

CBX5 has been reported to be overexpressed in gastric cancer tissues, promoting cell proliferation, migration, and invasion [137]. It interacts with the long non-coding RNA NEAT1 to enhance the stability of c-Myc mRNA, thereby facilitating gastric cancer progression, in vitro and in vivo [138].

Additionally, CBX5 has been shown to regulate the expression of DNA repair genes, contributing to genomic instability and chemoresistance in GC cells [60].

### Hepatocellular carcinoma

CBX1 has been shown to interact with the long non-coding RNA HOXD-AS1, forming a complex that regulates the expression of genes involved in HCC progression, including both in vitro *and* in vivo components. CBX3 promotes HCC cell lines proliferation by downregulating the expression of p21, a key cell cycle inhibitor [139, 140]. CBX5 has been less extensively studied in HCC than CBX1 and CBX3, but emerging evidence suggests its involvement in hepatocarcinogenesis. For example, recent findings have shown that CBX5 is overexpressed in human HCC tissues and it can be correlated with tumor growth and metastasis [141].

CBX1 is expressed at elevated protein levels in HCC, suggesting that patients exhibiting high CBX1 expression tend to experience poorer clinical outcomes [142].

### Lung adenocarcinoma

In LUAD, CBX1, CBX3, and CBX5 are significantly upregulated compared to normal lung tissue, suggesting a possible oncogenic function [53]. Mechanistically, studies in mouse models demonstrated that CBX3 enhances tumor cell proliferation and migration by regulating cell cycle progression, facilitating the G1/S phase transition, and modulating the p53 pathway [143]. CBX3 has been shown to enhance the stemness properties of LUAD cell lines, promoting the expression of cancer stem cell markers and c-Myc targets [53]. CBX3 and CBX5 have been found to promote tumor cell proliferation and migration in vitro and regulate the expression of various cytokines that influence the tumor microenvironment [6]. In contrast, the CBX1-specific role in LUAD is less well-defined, although it has been observed to be upregulated in LUAD along with CBX3 and CBX5 [65]. In lung cancer, the expression level of CBX3 mRNA was found to be elevated and correlated with reduced overall survival [144, 145].

### Prostate cancer

CBX1 showed elevated expression in PCa. This protein acts as a coactivator of AR, enhancing its transcriptional activity and collaborating to activate target genes essential for PCa cell survival and proliferation in vivo [146]. The CBX1-AR interaction promotes the growth and maturation of AR-positive cells, contributing significantly to the development of castration-resistant prostate cancer (CRPC).

It has been observed in PCa mouse models that c-Myc plays a crucial role in PCa progression through a complex regulatory pathway involving CBX3 and miR-451a. c-Myc directly binds to the E-box element in the CBX3 gene’s first intron, upregulating its expression. High CBX3 levels then promote H3K9 methylation at the miR-451a promoter, inhibiting its synthesis [147]. This cascade reduces miR-451a levels in PCa cells, which counterintuitively leads to decreased cell proliferation, increased apoptosis, and diminished tumor formation capacity [147].

Increased expression levels of CBX3 have been linked to poor prognosis in prostate cancer, and it has been identified as an independent prognostic biomarker [148].

Silencing CBX5 in NEPC cell lines significantly reduces its expression, impairing cell growth and triggering apoptosis. This loss disrupts regulatory mechanisms that promote proliferation and alters key transcription factors like AR, essential for maintaining the malignant phenotype of NEPC [149].

### Hematological disorders

CBX1 overexpression in diffuse large B-cell lymphoma (DLBCL), has been associated with resistance to common anti-tumor drugs, suggesting a potential role in therapy resistance in leukemia [150]. The RBMX/RBMXL1-CBX5 axis is essential for the survival and proliferation of AML. Notably, RBMX and RBMXL1, RNA-binding proteins elevated in AML patients, directly interact with and regulate the transcription of CBX5 mRNA. Interestingly, depletion of RBMX/RBMXL1 leads to a decrease in CBX5 expression in mice, compromising leukemia cell survival [151].

## Targeting CBX chromodomain for cancer therapy

Targeting CBX ChD is a potential strategy for modulating PRC activity through its reader function. However, the flexibility of the ChD and high homology among the CBX ChD provide significant obstacles to developing potent, selective, and cell-permeable CBX ChD inhibitors (CBXi). Small molecules targeting CBX ChD have only recently been designed, and their characterization in cells has shown promising results.

This finding will further enhance the identification of new molecules and advance them toward clinical applications. Although CBX inhibitors are not yet in clinical trials, promising research is underway to target CBX proteins in cancer. The development of safe and effective CBX inhibitors will require further studies to address challenges such as off-target effects, bioavailability, and specificity. Future clinical trials may focus also on combination therapies or targeted inhibitors of Polycomb complexes and their associated proteins. Table 3 summarizes the mechanism of action of the different CBX modulators.

**Table 3:** Mechanism of action of CBXs modulators.

Target Subunit	Ligand	Mechanism of action	Site of Action	Ref
CBX4/7	UNC3866	UNC3866 inhibits proliferation of PC3 prostate cancer cells.	Prostate cancer	[153]
CBX7	MS37452	It displaces CBX7 from the INK4A/ARF locus in prostate cancer cells, thereby de-repressing the transcription of p16/CDKN2A	Prostate cancer	[172]
CBX7	MS351	MS351 inhibits CBX7 binding to H3K27me3	Mouse embryonic stem cells. PC3 prostate cancer cells	[173]
CBX7	Compound 33 F	Developed through rational design to modify an L3MBTL1 methyllysine-binding inhibitor.	G-401 cells	[174]
CBX4/7	UNC4976	Allosteric modulation of CBX7 that abolishes its function as a reader of H3K27me3 marks and enhances its non-specific binding to DNA.	Prostate cancer. Embryonic Stem Cells (ESCs)	[152]
CBX6	Ligand 5	It binds to the beta groove of CBX6, which encompasses the lysine trimethylation binding pocket, along with a (-2) pocket and a hydrophobic cleft extending from the binding site.	Hepatocellular carcinoma (HCC)	[154]
CBX6/8	Ligand 22	Selectively binds to both CBX6 and CBX8 over other CBX ChDs.	Acute myeloid leukemia (AML)	[155]
CBX8	SW2_110A	It binds to the ChD of CBX8, preventing its association with chromatin, which leads to inhibition of proliferation and deactivation of MLL-AF9 target genes in THP1 leukemia cells.	THP1 leukemia cells	[155]
CBX8	UNC7040	Allosteric modulation of CBX8 that abolishes its function as a reader of H3K27me3 marks and increases its non-specific binding to DNA, leading to inhibited proliferation in lymphoma cells.	Diffuse Large B-Cell Lymphoma (DLBCL), CRC, ESCs	[157]
CBX2	SW2_152F	Selective CBX2 chromodomain inhibitor prevents and reverses neuroendocrine differentiation in prostate cancer cells.	Prostate cancer	[159]

### CBX4/7

The first CBX ChD ligands were peptides with the highest affinity for CBX7, Kd = 0.28 Å} 0.05 μM, and five-fold selectivity over CBX8 [152]. Subsequently, UNC3866 was derived, showing the same specificity for CBX7 and CBX4 but an elevated affinity than the first molecule; modification of the trimethyl lysine to dimethylglycine improved cell permeability. UNC3866 treatment induces a senescence-like phenotype in PC3 prostate cancer cells, inhibiting their proliferation at IC50 = 7.6 μM [153]. The derivative of UNC3866, named UNC4976, was modified with a sizable norcamphor group on the lysine instead of diethyls. This modification does not change the in vitro binding properties of the molecule but induces a conformational change, increasing the affinity of CBX7 ChD to DNA. Therefore, the enhanced efficacy UNC4976 results from the simultaneous antagonism to the H3K27me3-specific binding of CBX7 to target genes while increasing non-specific binding to DNA. This positive allosteric modulator (PAM) activity shifts the equilibrium of CBX7-containing PRC1 away from H3K27me3 target regions [152].

### CBX6

Peptidic ligand 5 selectively inhibits CBX6 ChD by binding to a small hydrophobic pocket close to the aromatic residues. It displays a nice affinity (Kd=900 nM) with an in vitro selectivity greater than 5-fold over the other CBXs. In addition, peptidic ligands 22 and 23, the first dual-selective inhibitors for CBX6 and CBX8, showed excellent affinity to CBX6 and CBX8. Lead compounds derived from this study demonstrated promising efficacy in reducing cell growth in rhabdoid tumor cell lines [154].

### CBX8

Using a selection process based on directed DNA-encoded libraries (DELs) against multiple ChDs led to the development of SW2_110A, which is a selective, cell-permeable inhibitor of the CBX8 ChD displaying a Kd of ~800 nM, and minimal 5-fold selectivity for CBX8 ChD over all other CBX paralogs in vitro (no binding to CBX4 and CBX6, 20- fold over CBX7, 5-fold over CBX2). SW2_110A explicitly inhibits the binding of CBX8 with chromatin and reduces the proliferation of THP1 leukemic cells carrying the MLL-AF9 translocation [155, 156]. Another study identified a potent PAM of CBX8 called UNC7040. This molecule can antagonize CBX8 binding to H3K27me3 while increasing interactions with DNA and the involvement in ncPRC1 [157, 158].

### CBX2

In vitro studies identified the CBX2i SW2_152F compound showing a Kd of ~80 nM, and 24 to 1000-fold selectivity for CBX2 over the other CBXs. This inhibitor blocks CBX2, inducing neuroendocrine differentiation in prostate cancer cells. This occurs mainly through the repression of AR signaling in neuroendocrine differentiated prostate cancer cells [159].

## Conclusions

CBX proteins are crucial in gene regulation, chromatin structure, and epigenetic modifications. Although CBX proteins have distinct, context-dependent functions, in general, CBX1 and CBX5 are involved in transcriptional repression and heterochromatin formation; CBX2 and CBX8 regulate the cell cycle and maintain stem cell properties; CBX3 ensures genomic stability and facilitates DNA repair; and CBX4 is essential for ubiquitin-mediated protein degradation. The dysregulation of CBX expression has been linked to various cancers, influencing tumor initiation, progression, and metastasis. The interplay of CBXs within the PRC1 significantly impacts cancer growth through complex regulatory mechanisms. CBX interactions and interconnections are essential in cancer research, as they provide insights into the complex molecular pathways that drive tumor development and progression. Thus, understanding CBX expression patterns and networks in cancer cells will be crucial for elucidating their roles in carcinogenesis and identifying potential therapeutic targets. While several drugs have been identified that selectively inhibit different CBX proteins, further research is needed to develop specific CBX modulators and assess their safety and efficacy in clinical settings. Specifically, the main challenge in developing new, specific modulators lies in the strong homology of their ChD domains, combined with the complexity of their roles in gene regulation and tumor biology. On the other hand, despite these challenges, there has been some success: recently, potent and partially selective inhibitors targeting CBX2, CBX4, and CBX7 residues have been developed showing interesting anticacer properties. These findings emphasize the need for further research that will first fully clarify the molecular roles and mechanisms through which CBXs contribute to cancer hallmarks in specific cellular and tissue contexts and, second, identify novel, potent inhibitors with improved specificity and efficacy, aiming to advance them into clinical applications to enhance patient care.
